# A patient-centred approach to embryo donation for research

**DOI:** 10.1186/s13584-016-0098-z

**Published:** 2016-11-07

**Authors:** Catarina Samorinha, Susana Silva

**Affiliations:** ISPUP-EPIUnit, Universidade do Porto, Rua das Taipas, no. 135, 4050-600 Porto, Portugal

## Abstract

Most couples enrolled in Assisted Reproductive Technologies’ (ART) treatments need to make decisions regarding embryo disposition, as they are asked to sign a consent form about embryo donation for research. Beyond the current assessment of patients’ individual experiences and levels of satisfaction with care delivery, we argue that it is crucial to provide stakeholders in health systems with feedback on patients’ views about legal and regulatory frameworks. Such knowledge will lend credence and robustness to the consent that the couples give, and will contribute to the implementation of informed relational ethics in clinical practice and to improved patient-centredness in the field of ART.

## Background

The legal and regulatory landscape on human embryo research varies among countries [1]. Taking into account the 58 countries with coherent data on national policies or guidelines regarding research on human embryos in three main sources of information [2–4], 22 countries ban such research, and 19 countries only allow research with surplus in vitro fertilization (IVF) embryos, prohibiting the creation of embryos only for research purposes. Six countries permit the creation of human embryos for research aims, four allow research only on imported embryos and the remaining seven countries have no legislation on human embryo research.

In countries permitting research on human embryos, Assisted Reproductive Technologies’ (ART) organizations and practitioners are increasingly being required to obtain consent from the woman and the man stating that their embryos can be used in scientific studies [4–6]. Thus, most couples enrolled in ART treatments need to make decisions regarding embryo disposition, as they are asked to sign a consent form about embryo donation for research.

Evidence has consistently shown that this is a complex and difficult decision-making process, involving several stages and patient preferences that can change over time [7–10]. The paper from Aviad Raz and colleagues, which was recently published in the IJHPR, provides an extended approach to the understanding of the attitudes, values and perceptions of IVF patients who decided to donate cryopreserved embryos to research, based on in-depth interviews with patients from an IVF unit in Israel [11]. In accordance with previous studies, that paper shows how decision-making about embryo donation for research is influenced by two main iterative and dynamic dimensions: patients’ hierarchical structuring of the possible options regarding embryo disposition, framed on patients’ beliefs about what should be done (considering for example that it is better to donate than waste the embryos) [8, 12]; and patients’ representations about the moral, social and instrumental status attributed to embryos, as for example, considering the embryo as a child or a life [8, 13] or as a valuable resource that did not yet have a human identity [11]. It also highlights how regulations constrain individuals’ choices.

We call for a renewed debate on embryo donation for research that goes beyond the current assessment of patients’ individual experiences and levels of satisfaction with care delivery [14, 15]. This debate should also reflect on the analysis of real circumstances under which decisions about embryo disposition are being made - including psychosocial and reproductive factors, and structural drivers (such as norms and values within society; global and national economic and sociolegal policy; processes of governance at the global, national, and local level; and health care system characteristics) [16]. With the purpose of enriching patient-centredness in embryo donation for research, this commentary will discuss two main issues regarding patients’ views about legal and regulatory frameworks. The first issue concerns the circumstances under which informed consent should be delivered, explained and signed, considering that patients’ attitudes about embryo disposition evolve over time. The second issue concerns the patients’ perceptions about storage limits for embryos, taking into account that they may shape decision-making on embryo disposition and that there is no evidence justifying the current storage periods.

### Timing set to obtain consent

There are differences between countries regarding whether the informed consent forms regarding embryo donation for research should be signed prior to the first treatment [17], during treatment [18] or after treatment is completed [19]. Evidence showing changes in couples’ willingness to donate embryos for research supports the idea of a two/three-stage process to obtain full informed consent [9, 10, 20]. It also reinforces the argument that it should be signed only after the infertility treatment is completed, as recommended by the Ethics Committee of the American Society for Reproductive Medicine [19].

There is a wide debate about the meaning of informed consent and what is needed to guarantee its legitimacy and validity [21, 22], in a context where the relationship between freedom of action and choice, on the one hand, and the influence of medical expertise and advice, and the social context, on the other, emerge as an important topic of reflection [22]. Overall, it is important that the consent is effectively informed (which requires an understanding of its content as well the comprehension of the oral and written information provided), voluntary (without any pressure or coercion, external or internal, concerning decision-making) and reflected (preceded by time to think about the decision) [5, 23, 24]. These elements are especially relevant when the decision process involves the search for consensus among partners [25].

Silva et al. [26], for example, also discuss the conditions under which informed, voluntary and reflected consent is conceptualized and implemented. They have suggested that informed consent should not be seen as the result of a purely rational and autonomous process of decision-making, based on a deep assessment and understanding of the information provided by health professionals, as it is also guided by feelings of trust in these professionals [22]. Interactions and relationships can enhance, as well as restrict, the autonomy to consent [27]. Consent is regarded as a perceived and experienced process constructed through interactions between individuals and their social contexts, where emotions, desires and feelings shape patients’ responses and decision-making. It is conceived as an ongoing process rather than as a discrete act of choice taking place in a given moment of time [20], which calls attention to the importance of being revocable at any time.

The process of informed consent may constitute an opportunity for humanization, democratization, accountability and transparency of processes and decisions [19, 28] concerned with ART by fostering dialogue and trust between health professionals and patients [29, 30] and providing a space for reflecting about cryopreservation and decision-making regarding embryo donation for research. However, it can also be reduced to a formality that may be guided by legitimate medical strategies to manage risks, expectations and responsibilities in the field of ART [30, 31].

Thus, attention must be drawn to the need to promote an in-depth analysis of the clinical, social and political contexts that influence the consent process [21], including the relational and interactional aspects [22], the changes over time due to fluctuations in the information exchanged with the health professionals, and variations in the social networks or in the reproductive trajectories of patients [9, 20]. The responsibility of scientific and medical institutions, health professionals and researchers regarding the provision of accurate and timely information that is attentive, responsive, and tailored to patients’ needs should be highlighted, in a context where a decrease in patients’ willingness to donate embryos for research over time is being observed [10, 20]. Investment in information provision is especially important, taking into account that the majority of patients highlighted feelings of trust and reciprocity towards the health professionals who contacted with them, as well as confidence in the medical and scientific institutions.

### Length of embryo storage

There are regulatory differences regarding the maximum length of time for embryo storage worldwide [32], which may influence cross-border reproductive care services [33, 34]. Embryo storage limits are 3 years in Portugal, 5 years in Denmark, Egypt or Norway and 10 years in Austria, Australia or Taiwan [4]. It can be longer in some countries, such as the United Kingdom, where a maximum storage period of 55 years is available [35], and it is unlimited in Canada and Finland [4].

The establishment of a storage limit for embryos, up to now, has relied mostly on social and political criteria [36, 37]. In fact, the impact of long-term storage on children’s and parents’ health and well-being is still poorly known [38]. Additionally, evidence consistently shows that the storage length does not detract from the quality of cryopreserved embryos [38, 39]. In this context, knowledge about patients’ views regarding the embryo storage limit is necessary [32].

Guidelines to regulate applications to extend embryo storage should have more flexibility and sensitivity to take into account the life conditions of patients and their reproductive trajectories [32]. Moreover, gaps and misconceptions in awareness of cryopreservation were found, suggesting that more information should be provided to patients concerning embryo cryopreservation, and namely about the storage periods [32, 40]. In this context, the provision of accurate and adequate information regarding policies on embryo storage and the development of consensual guidelines on storage limits may help raise awareness about cryopreservation among patients and also health professionals.

## Conclusions

It is now widely recognized that high quality infertility care comprises more than just the effectiveness of care [41, 42] and should be patient-centred [43–45]. However, existing studies adopting a patient-centred approach do not explore the specific process of decision-making about embryo disposition, and in particular regarding embryo donation for research. Knowledge of patients’ perspectives and experiences with regard to embryo donation for research is essential for the conceptualization of patient-centred policies and for ethics in clinical practice at the following levels:

- To analyse openness and information about research with human embryos;
- To sustain stakeholders’ decisions regarding the suitability of research projects using cryopreserved embryos; and
- To disseminate ethically robust evidence to inform policies and guidelines on embryo cryopreservation and embryo disposition, namely concerning the informed consent implementation on a two/three-stage process [9, 10, 20], and the establishment of storage periods and the reasons for limitations in these periods [32], in a context where the views of the patients apply across legal and political boundaries.

Thus, this commentary calls for a renewed debate that includes the views of patients about the legal and regulatory contexts that frame the clinical practice.

Further research needs to be carried out regarding the meanings attributed by IVF couples to the possibility of visualizing their cryopreserved embryos and how these meanings influence decision-making on embryo donation for research. The opinions of IVF patients about what should happen when there is no agreement between partners concerning embryo disposition needs to be assessed. Listening to clinic staff perspectives and experiences is required to achieve an integrated view about the human and system factors that influence patient-centred care. It is also crucial to analyse egg and sperm donors’ perspectives on legal and regulatory frameworks as well as the donors’ real-world decisions about the disposition of the embryos resulting from their own gametes.

## Abbreviations

ART: Assisted reproductive technologies
IVF: In vitro fertilization
